# Inflammation and Alzheimer's Disease: Mechanisms and Therapeutic Implications by Natural Products

**DOI:** 10.1155/2021/9982954

**Published:** 2021-08-02

**Authors:** Mashoque Ahmad Rather, Andleeb Khan, Saeed Alshahrani, Hina Rashid, Marwa Qadri, Summya Rashid, Rana M. Alsaffar, Mohammad Amjad Kamal, Muneeb U. Rehman

**Affiliations:** ^1^Department of Biochemistry and Biotechnology, Annamalai University, Annamalai Nagar, Tamil Nadu 608002, India; ^2^Department of Pharmacology and Toxicology, College of Pharmacy, Jazan University, Jazan 45142, Saudi Arabia; ^3^Department of Pharmacology & Toxicology, College of Pharmacy Girls Section, Prince Sattam Bin Abdulaziz University, P.O. Box 173, Al-Kharj 11942, Saudi Arabia; ^4^King Fahd Medical Research Center, King Abdulaziz University, P. O. Box 80216, Jeddah 21589, Saudi Arabia; ^5^West China School of Nursing/Institutes for Systems Genetics, Frontiers Science Center for Disease-related Molecular Network, West China Hospital, Sichuan University, Chengdu 610041, Sichuan, China; ^6^Enzymoics, 7 Peterlee Place, Hebersham, NSW 2770; Novel Global Community Educational Foundation, Australia; ^7^Department of Clinical Pharmacy, College of Pharmacy, King Saud University, Riyadh 11451, Saudi Arabia

## Abstract

Alzheimer's disease (AD) is a neurodegenerative disorder with no clear causative event making the disease difficult to diagnose and treat. The pathological hallmarks of AD include amyloid plaques, neurofibrillary tangles, and widespread neuronal loss. Amyloid-beta has been extensively studied and targeted to develop an effective disease-modifying therapy, but the success rate in clinical practice is minimal. Recently, neuroinflammation has been focused on as the event in AD progression to be targeted for therapies. Various mechanistic pathways including cytokines and chemokines, complement system, oxidative stress, and cyclooxygenase pathways are linked to neuroinflammation in the AD brain. Many cells including microglia, astrocytes, and oligodendrocytes work together to protect the brain from injury. This review is focused to better understand the AD inflammatory and immunoregulatory processes to develop novel anti-inflammatory drugs to slow down the progression of AD.

## 1. Introduction

Alzheimer's disease (AD) is a slow, fatal, human neurodegenerative disorder depicted by the progressive loss in memory retrieval, learning, language, and other cognitive activities. Two main toxic misfolded protein fragments including amyloid plaques and neurofibrillary tangles begin to assemble inside the brain, which sequentially and moderately accelerates the advancement of the disease and eventually leads to neuronal dysfunction and cell death. Amyloid-*β* lesions and neurofibrillary tangles slowly obliterate the hippocampus, and forming new memories becomes more complex. Several etiological assumptions have been projected for AD, such as genetic abnormalities, the extent of neurofibrillary tangles, irregular APP processing, a discrepancy of neurotrophic factors, mitochondrial dysfunction, microelement neurotoxicity, impairment in energy metabolism, and oxidative stress [1]. AD leads to neuronal dysfunction and cognitive deficits by the accumulation of the amyloid-beta peptide (A*β*) in the human brain [2]. Short-term memory, visuospatial dysfunction, and praxis are the most common symptoms of AD. Generally, inflammation is considered to be an intricate defense mechanism that appears internally in response to agitated and altered homeostasis. Neuroinflammation predominantly illustrates the inflammatory responses taking place in the central nervous system (CNS) which includes well-balanced innate and adaptive immune systems. Neuroinflammatory mechanisms immensely facilitate the development of the brain as well as in the neuropathological episodes. Cell-autonomous and non-cell-autonomous operations are the two separate pathological operations involved in neuronal death in AD as well as in most neurodegenerative diseases. The cell-autonomous procedure involves the occurrence of intrinsic impairment in the degenerated neurons which triggers their extinction, whereas the non-cell-autonomous operation involves the divergent deflation of the afflicted neurons stimulated by pathological interrelations with neighbouring cells, including astrocytes and microglial cells or immune cells such as macrophages and lymphocytes impregnated from the periphery.

The defensive mechanisms against various toxins, injury, and infection caused by several other means lead to the interruption in the expression of proinflammatory and anti-inflammatory cytokines and initiate chronic neuroinflammation [3, 4], which in turn triggers the liberation of various cytokines and the stimulation of microglial cells. Researchers postulated that continuous immune response is linked with neurodegeneration as well as the acceleration of both A*β* and NFT lesions and supports the insight into initial incidents of A*β* lesions followed by the progression of NFTs [5, 6]. Extensive studies have been carried out to distinguish the mechanisms involved in the progression and prevention of these two abnormal lesions of AD such as A*β* and NFT. However, there is no such treatment accessible that can effectively change both the pathologies involved in the development of AD [7]. Studies have investigated the response of inflammatory markers both in the postmortem and preclinical samples in AD patients, and they have suggested that neuroinflammation is the main and early feature of AD, which actively contributes to the pathogenesis of AD [8, 9]. In this review, we summarize the role and the mechanism of inflammation in AD and the therapeutic manipulation against the progression of AD.

## 2. The Baseline of Neuroinflammation in AD

Amyloid precursor protein (APP) is a glycosylated intermembrane protein, ubiquitously found in numerous tissues and abundantly found in the brain, that undergoes enzymatic cleavage and produces A*β*1-40 and/or A*β*1-42. APP functions as a trophic factor, which plays a substantial role in synaptogenesis, neurite outgrowth, and remodeling of synapses [10]. During neuronal differentiation and maturation as well as in the pathological events of AD, the expression of the APP protein gets subsequently increased [11]. The production of A*β*40-42 residues tends to aggregate into fibrils and deposit with age. A*β*1-42 is longer, hydrophobic, and fibrillogenic that is less produced than A*β*1-40. It is the principal species deposited in the brain [12, 13] and favors oligomerization and subsequent fibril formation [14, 15]. A*β*1-42 is deposited as the main component of senile plaques, and these oligomers are collectively produced by the activities of neurons and related astrocytes [16]. A*β* peptides are produced abundantly during the development of AD and start to accumulate in the brain. These oligomers subsequently destabilize microtubule-associated tau protein, which leads to its hyperphosphorylation and initiates its accumulation to form filaments in the neuron. Due to the destabilization of tau protein, the skeleton of neurons begins to degenerate and the communication between the neurons is lost [17]. It has been investigated in various studies whether senile plaques and NFTs are the only abnormalities involved in neuronal damage. Both imaging and postmortem studies showed that treatments for AD patients attenuated the A*β* pathology but could not alter its progression [18]. Thus, it has been suggested that apart from A*β* and NFTs, other factors are actively involved suggested that, apart from A*β* and NFTs, several other factors are also actively involved in the impairment of neurons in AD. Inflammation is the prime factor involved in the progression of various diseases including neurodegenerative diseases, which is validated by the increase in the expression of proinflammatory cytokines in the brain tissues and the blood samples of AD patients [19, 20]. Also, it gets expressed from the events of activated specialized macrophages such as microglia and astrocytes around the A*β* aggregates in AD [18].

Neuroinflammation also plays an active role in other neurodegenerative diseases, as indicated by the overexpression of proinflammatory markers, and is the target for therapeutic manipulations [21]. Several studies have proposed that inflammation triggers the increased accumulation of A*β* peptides, and the activated astrocytes and microglial cells are involved in the deposition of oligomeric peptides [22]. Several pro- and anti-inflammatory cytokines have been identified, in which proinflammatory cytokines are involved in the promotion of inflammatory responses, whereas anti-inflammatory cytokines trigger the regulatory responses to control the neuronal damage caused by proinflammatory cytokines. Several protein kinases such as glycogen synthase kinase 3*β* (NF-*к*B), mitogen-activated protein kinase (MAPK), cell division cycle 2 kinase (cdc2), and JAK-STAT are involved in AD progression. Thus, these kinases may be activated by elements of AD pathology such as inflammation, oxidative stress, A*β*, and cell cycle reentry [23]. Caspases are also known to play a significant role in inflammation; for example, caspase-1 plays an essential role in the maturation of proinflammatory cytokines, and other caspases such as caspase-3, caspase-7, and caspase-8 may regulate the activation of microglial cells [24]. Chemokines are also important factors that play an essential role as mediators in inflammatory processes. During inflammatory events in CNS, enhanced chemokines production and initiation of microglial and astrocyte chemotaxis takes place. Astrocytes and microglial cells perform significant activities in homeostasis and brain functions and are the main agents involved in the inflammatory processes in AD [25]. It is indicated that activation of microglial cells may be an early event in the pathogenesis of AD and may trigger the disruption of synaptic transmission and encourage early memory dysfunction [26]. In response to various toxins, astrocytes and microglial cells get activated and lead to the process called reactive gliosis, which significantly contributes to the pathogenesis of neuroinflammation. In the reactive gliosis process, an astrocyte-specific intermediate filament protein (GFAP) plays a significant role in the homeostasis of CNS, and allograft inflammatory factor-1 (Alf1), a microglial specific protein, is found to be increased in an experimental study of AD [27]. The further inflammatory cascade is validated by the exaggeration of astrocytes and glial cells and the overexpression of proinflammatory cytokines in the AD brain [27]. Proinflammatory cytokines emphasize oxidative stress through the activation of NF-*κ*B, and these transcription factors are the key players in the process of neurogenesis as well as in other physiological processes [28], and are involved in the process of synaptic plasticity [29]. Some assert that AD-affected brain regions contain enhanced levels of neuroinflammatory mediators via the increased inflammatory responses [30].

### 2.1. Effect on Cells of Inflammatory Response in Aged AD Brain

In different tissues, transcriptional variability in between cells including the hematopoietic cell lineage gets upregulated during aging [31, 32]. Also, a reduction in the population of naive T and B cells occurs, whereas terminally differentiated T cells increase in number in aged persons [33, 34]. Memory B cells are attenuated in aging, which leads to the reduced antibody response, while in aged humans and aged mice, mature B cells, termed age-associated B cells, may trigger autoimmunity and inflammation [35, 36]. In aging, an inefficient immune response makes the person more sensitive to various infections and autoimmune disorders, which are associated with and eventually leads to the deterioration in cognitive functions. During senescence, cells begin to assemble inside the body by accessing cell cycle arrest stimulated by stressors. It has been elucidated that an intricate mechanism is involved in the biosynthesis of these cells and in the significant role they play in various physiological and pathological events [37, 38]. The association of cells during senescence in host immunity is parallel to their potential to extrude proinflammatory cytokines known as senescence-associated secretory phenotype (SASP) [39]. NF-*κ*B plays a significant role in the initiation of SASP which is triggered by various events such as stress, DNA damage, and developmental indications, which in turn promote the transcription of IL-6, IL-8, IL-1*β*, and TNF-*α* inflammatory markers [31]. Though microglia play an important role in the augmentation of neuroinflammatory responses and the early occurrence of A*β* oligomers, microglia begin to work against these toxic peptides by producing proteolytic enzymes that help to clear and destroy A*β* fibrils, initiate inflammatory responses, and assist in tissue homeostasis [40, 41]. In the normal functioning of the immune mechanism, activated microglia and the cytokines help to clear the foreign agents and rejuvenate the damaged tissues. The formation of A*β* continues during AD which leads neuroinflammation, which may instigate a vicious cycle that further leads to A*β* generation and alter the functioning of microglial cells [42, 43].

#### 2.1.1. Microglia

Microglia, being the prime innate immune cells of the brain, play an indispensable role in the homeostasis of the CNS. They form and trigger the first line of defense inside the brain, thereby countering the pathological incidents through an array of inflammatory responses. Inflammatory cytokines, chemokines, and other markers are conferred to stimulate the activation of microglial cells, which potentially support injury and neuronal dysfunction. It is documented that APP, amyloid aggregates, and fibrils are potent glial activators, which stimulate an inflammatory cascade and the release of microglial neurotoxic cytokines [44]. Autopsies of AD brain patients exhibited reactive microglia adjacent to A*β* plaques; this has been explicated that A*β* triggers several pathways such as the NF-*κ*B-dependent pathway, mitogen-activated protein kinase (MAPK) pathways, the cell surface binding of microglia, and the initiation of extracellular signal-regulated kinase to stimulate proinflammatory gene expression [25, 45]. The A*β* peptides are known to incite NADPH-intervened priming in microglia, which contributes to ROS generation that leads to neurotoxicity [46].

Several interleukins, including IL-2, IL-6, and IL-1*β*, tumor necrosis factor (TNF-*α*), and anti-inflammatory transforming growth factor*β*1 (TGF*β*1) were found in enhanced levels in AD subjects [25, 47], and the explication of the postmortem samples manifested the higher amounts of IL-1*β*, interferon-*γ* (IFN-*γ*), TNF-*α*, and NOS and the generation of free radicals. These observations further support the inclusion of microglial cells in the stimulation of an inflammatory cascade signifying the occurrence of several AD phenotypes and discrete function during the advancement of the disease. Several experimental investigations have been carried out on blood samples and cerebrospinal fluid (CSF) samples in AD patients, and these samples revealed enhanced expressions of proinflammatory markers including IL-2, IL-6, IL-1*β*, TNF-*α*, and IL-10 anti-inflammatory markers [48]. Probing of CSF has come up with new hope in providing essential information for the detection of unknown neurodegenerative and neuroinflammatory biomarkers, as it reflects more precise pathological and metabolic modifications in the CNS compared to other fluids.

#### 2.1.2. Astrocytes

Besides microglia, astrocytes have also been found to participate in the synaptogenesis, neurotransmission, BBB constancy, and neuropathology of AD, which induces the expansion of proinflammatory cytokines [49]. These cytokines instigate secretases which support the A*β* generation from APP, and the reactive astrocytes contain BACE, an A*β* cleavage enzyme which plays a crucial role in A*β* production, and presenilin-1, which also favors A*β* production by releasing *γ*-secretase that cleaves the APP enzyme to give to A*β* oligomers. In the CNS, astrocytes not only play an indispensable role in A*β* production, but they also participate in the degradation of A*β* and form a protective barrier in between neurons and toxic peptides [50]. Astrocytes play an essential role in imparting neuronal development and synaptic plasticity in the CNS, energizing the neurons, and maintaining brain homeostasis. Elevated amounts of calcium-binding protein S100b are primarily represented by astrocytes which act as a cytokine, which has been reported in the postmortem studies of AD patients. Levels of inducible nitric oxide synthase (iNOS) may get upregulated by S100b, resulting in the initiation of cyclooxygenase-2 (COX-2) in microglial cells and leading to the enhanced expansion of nitric oxide (NO) and superoxide radicals, which in turn may cause direct or indirect death of neurons. The C-terminal 100 amino acids of bAPP in AD pathology plays a crucial role in the instigation of astrogliosis and the death of neurons [47]. Microglia are involved in the phagocytosis and degradation of A*β* oligomers, while astrocytes play an essential role in the clearance and deterioration of these toxic peptides [51]. A study postulated that astrocytes in the cortex compile A*β*42 fragments which imperatively leads to the enhancement of A*β* pathologies, which confirms that astrocytes contribute to extended neuroinflammation, expressing iNOS that facilitates NO-induced toxicity [52]. Further, the appearance of active astrocytes in AD patients and AD animal models confirm the prominent role of these supporting cells of the brain in neurodegenerative diseases [51].

#### 2.1.3. Oligodendrocytes

**Figure 1:**
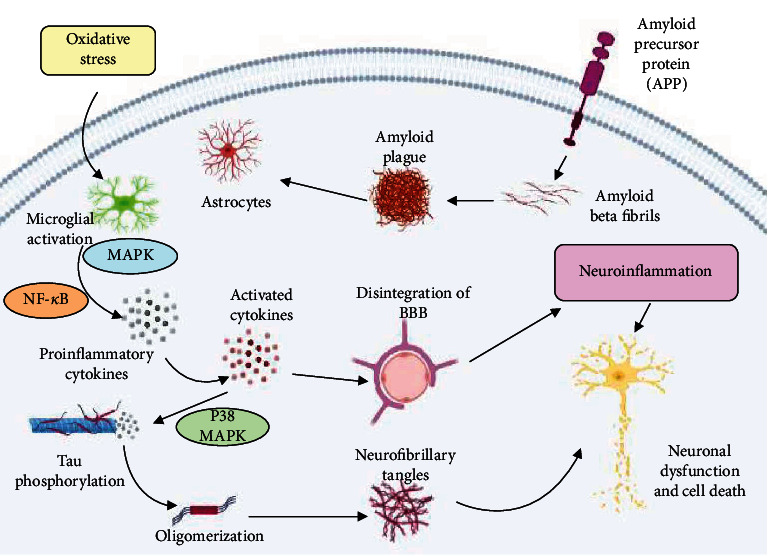
A*β* fibrils lead to neuronal death, which include ROS generation, neurotoxicity, release of inflammatory cytokines, and activation of the complement system. Due to the accumulation of A*β* oligomers, neuronal degeneration may stimulate the microglial activation, which will initiate the liberation of proinflammatory mediators, neurotoxins, and free radicals but also play a pivotal role in the elimination of A*β* peptides. These peptides trigger oxidative stress and promote inflammatory processes in neurons, which enhance the production of A*β* peptides via increased APP expression. Activated MAPK (a mitogen-activated protein kinase) and NF-*к*B (nuclear factor kappa-light-chain-enhancer of activated B cells) lead to the production of proinflammatory cytokines, and their increased expansion promotes APP processing and disintegration of BBB (blood-brain barrier) and aggravates the phosphorylation of Tau protein and eventually leads to the formation of neurofibrillary tangles via the activation of p38-MAPK which leads to neuronal degeneration (created with http://BioRender.com/).

Oligodendrocytes are the crucial cells for neurotransmission and are the myelin-forming cells in the CNS. The oligodendrocytes together with the myelin sheath support the CNS and form an envelope around the axons, which act as an insulator and initiate smooth transmission of signals in between neurons [53, 54]. Studies manifest that improper functioning of oligodendrocytes may trigger the pathophysiology of diseases such as bipolar disorder, schizophrenia, AD, PD, and several other neurodegenerative disorders [55–58]. Several studies reported that pathological lesions and demyelination in AD white matter and A*β* fibrils in the grey matter have been substantially manifested in sporadic, familial, and mouse models of AD [59, 60]. Oligodendrocytes are involved in the modulation of ion homeostasis, and oligodendrocyte precursor cells (OPCs) are the specific cells that take part in the formation of synapses with glutamate neurons in the cortex and hippocampus, as well as in other areas of the brain [61–63]. We could speculate that neuronal AD pathology, which is characterized by neuronal and axonal dysfunction, could alter the amounts of myelin produced by oligodendrocytes. Thus, this dynamicity of myelin and oligodendrocytes could ultimately affect behavior. Several studies demonstrate that A*β* oligomers are involved in the process of demyelination and the loss of oligodendrocytes and having less antioxidant and iron content; these cells are more susceptible to oxidative stress, which is one of the main protagonists in inflammation [64, 65]. Also, inflammation is associated with activated oligodendrocytes in AD conditions [66]. Thus, inflammation, myelination, and demyelination are closely associated with oligodendrocytes (Figure 1).

### 2.2. Inflammatory Response Pathway Mechanisms in AD and Their Potential Therapeutic Targets

Composing the exact descriptions of AD is still underway, and continuous efforts are being made to reduce the progression of the disease. Presently, there are no treatments available to control cognitive impairment. Excessive production and the accumulation of A*β*-amyloid and hyperphosphorylated tau protein are the two main events that are considered the principal cause of AD. However, excessive A*β* generation may subsequently lead to immune dysfunction rather than the advancement of the disease itself. Given this, several pathways such as the NF-*к*B, MAPK, and JAK-STAT pathways are engaged in innate immunity and are linked with the expansion in AD [67, 68]. Several studies have investigated the possible mechanisms of these pathways and targeting therapeutic drugs for the inflammatory conditions in AD, which provide promising results against AD. Efforts are constantly being implemented to comprehend the AD pathophysiology and the involvement of several kinase pathways in the process of memory impairment and cognitive dysfunction. Therefore, in the following segments, we will emphasize the role of NF-*к*B, MAPK, and JAK-STAT pathways in support of the mechanisms associated with AD pathology.

#### 2.2.1. NF-*к*B Pathway

Activation of microglial cells results in the formation and release of proinflammatory cytokines, which trigger neighbouring astrocyte neurons to generate further production of A*β*_1-42_ oligomers, which may promote the upregulation of neuronal cell death by further activating inflammation [16]. Several therapeutic drugs are being used to inhibit neuroinflammation linked with disease progression, which reveals positive effects through various mechanisms such as maintaining Ca^2+^ homeostasis, inhibition of cyclooxygenase (COX), and targeting *γ*-secretase [69]. Several studies reported that toxic agents were found to be involved in the activation of regulatory inflammatory marker NF-*κ*B by triggering the degradation of inhibitor kappa B (I*κ*B) [70]. Overexpression of TNF-*α* plays an essential role in the activation of transcription factor NF-*κ*B [71]. Proinflammatory cytokines emphasize oxidative stress by provoking expression of the iNOS gene via activation of NF-*κ*B. The NF-*κ*B factors play a key role in the CNS in several physiological processes associated to signal transmission, cognition, and memory [29]. Dysfunction of the CNS, oxidative stress, and neuroinflammation are the major events in AD which are activated and progress via activation of NF-*κ*B. Generation of ROS triggers the IKKb enzyme which phosphorylates the heterodimer of NF-*κ*B, an inhibitor of kappa B (I*κ*B) causes its degradation by the ubiquitin-proteasome pathway, and the detachment of I*κ*B from the dimer initiates the influx of NF-*κ*B into the nucleus.

NF-*κ*B acts as a prime factor of innate immunity in environmental and genetic risk factors in different AD models [72]. NF-*κ*B signaling promotes BACE1 gene expression and A*β* processing and is considered to be the novel mechanisms essential for the advancement of AD [72]. Also, it has been reported by several studies that A*β* induces the enhanced expression of NF-*κ*B which in turn facilitates the expression of chemokines and cytokines, and these proinflammatory markers promote the progression of AD. In addition, activation of NF-*κ*B plays a crucial role in upregulating the levels of various microRNAs in the brain which further causes the diminishing of regulating proteins including synapsin-2, tetraspanin-12, and complement factor H in the CNS. Diminished extents of synapsin-2 lead to the dysfunction in synaptogenesis inside the neurons, whereas the declined proportion of tetraspanin-12 and complement factor H promote A*β* accumulation and initiate an inflammatory response in the neuronal cells [73]. Therefore, several neurological diseases are pathologically associated with the activation of NF-*κ*B. The NF-*κ*B pathway is supposed to play a significant role in regulating the cellular fate in a broad array of physiological and pathological conditions, which provides an opening to exploit its essential functions. Several other factors such as phosphorylation and degradation of I*к*B, DNA interaction, and its translocation are found to be associated with the activation of NF-*κ*B, which supports its compatibility for drug intervention. Numerous therapeutic agents have been implicated and have exhibited promising results in modulating the activation of NF-*κ*B in the CNS and attenuating the processes which initiate the decline of neurons. Polyphenols, antioxidants, and several other drug categories have been used and are usually preferred as these therapeutic agents claim specific targets.

Antioxidants, polyphenols, and nonsteroidal anti-inflammatory drugs (NSAIDs) have been found to inhibit the activation of NF-*к*B and have played an instrumental role in attenuating the A*β* burden [74]. Curcumin, a polyphenolic compound, has been widely studied and has been proven to be a potential therapeutic and neuroprotective agent which significantly attenuated the neuronal death and alterations in brain tissue by regulating the NF-*к*B pathway [75]. Several studies reported that plant-derived compounds significantly downregulated the expression of NF-*к*B and mitigated the neuroinflammation in cellular models of AD [76, 77]. Immense efforts have been instigated in understanding the mechanism of the progression of the disease and the efficacy of novel therapeutic agents for AD. Several therapeutic approaches have been employed to regulate the disease progression and attenuate the cognitive deficit in AD subjects. Researchers have recently proposed that decreasing the expression of genes associated with AD may play an essential role in diminishing toxicity caused by misfolded A*β* and Tau proteins which may attenuate the advancement of AD [78]. siRNA gene silencing is one of the therapeutic approaches employed in AD subjects to attenuate disease progression. Several studies have revealed that siRNA silencing may play an essential role in regulating the expression of genes and in silencing AD-associated genes such as APP, Tau, and BACE1 genes. Thus, it exhibited positive results in diminishing mRNA levels of target genes, which confirms the efficacious applications of the siRNA approach in AD. [79–81]. Several other studies also confirmed that the APP gene silencing in the transgenic mouse model may attenuate the A*β* extents and may significantly improve the cognitive dysfunction in mice, which further confirms the advancing role of siRNA gene silencing and is considered a potential therapeutic approach for AD pathology [82, 83].

#### 2.2.2. MAPK Pathway

These protein kinases are intracellular enzymes that make them feasible for cells to respond from the external environment; for example, inflammatory cytokines or osmotic shock triggers GTPase-dependent activation of several kinases. These kinases are involved in the phosphorylation and activation of MAPK kinases, which further phosphorylates and activates p38 MAPK and results in various adaptive responses. The p38 MAPK activation initiates the enhanced production of cytokines via direct phosphorylation of transcription factors and enhanced mRNA translation which code for proinflammatory cytokines [84]. In AD, postmortem studies have reported that exaggerated immunoreactivity of phospho-p38 MAPK was linked with A*β* plaques and neurofibrillary tangle-oriented neurons, and it was further observed in patients with AD that activation of p38 MAPK begins at the early stage of the disease [85, 86]. The upstream activator of p38 MAPK includes MKK6 that is in higher levels in postmortem brains of AD, which exhibits an active role of p38 MAPK in the advancement of the disease [67]. Activation of MAPK is the crucial factor for the inflammation event in the brain and has been investigated in several in vitro studies. A study showed that p38 MAPK leads to the activation of glial cells, in which both p38 MAPK and Erk cascades play an indispensable role in the transcriptional and posttranslational regulation of iNOS and TNF-*α* gene expression in activated glial cells [87]. The MAPK pathway has been found associated with amyloid-*β* fibrils and directs the transcription of inflammatory markers. Amyloid-*β* fibrils in microglia trigger brief and quick p38 MAPK activation, which results in the expression of inflammatory genes and enhances expression of proinflammatory cytokines [88]. In addition, it has been documented that microglial activation by APP mostly occurs via the p38 MAPK pathway, which initiates the generation of proinflammatory markers [89], and also it has been observed in an animal study that microglial MAPK signaling is involved in A*β*-induced neuroinflammation [90]. However, the p38 MAPK signaling pathway also plays a major role in the detrimental functions of activated astrocytes. Experimental data confirms that the release of IL-1*β* from microglia affects astrocytes, which leads to the activation of the NF-*к*B pathway [91], and IL-1*β* has been found to play a role in the activation of the MAPK pathway both in animal and human astrocytes [87, 92]. Though p38 MAPK leads to the production of iNOS and TNF-*α*, which causes a chronic inflammatory event, the regulatory function of the p38 MAPK cascade has been validated via the IL-1*β*-dependent inflammatory aggravation navigated by primary astrocytes. Also, it has been confirmed that regulating the activity of p38 MAPK causes the obliteration of the transcriptional activity of NF-*к*B and diminishes the expression of the NF-*к*B-dependent gene iNOS [93]. Several observations have shown new features in the p38 MAPK cascade are associated with AD pathology and extend all the way to designating p38 MAPK pathway inhibitors as therapeutic targets for the prevention of AD pathologies. Extracellular A*β* aggregates initiate and enhance MAKK6 which phosphorylates p38, which in turn initiates tau hyperphosphorylation, proceeds to neuronal apoptosis, and regulates synaptic plasticity. All these events are associated with the activation of p38 MAPK. Neprilysin, a cleavage enzyme involved in the degradation of A*β*, is also known to be afflicted by activation of p38. A study showed that SXFAD mice deficient in p38*α*, and the knockdown of p38*α*, leads to enhanced A*β* degradation by increasing the expression of neprilysin, a metalloproteinase in glial cells [94]. The activating transcription factor 4 (ATF4) is another factor that enables the activation of proapoptotic proteins of p38 by initiating MKK6 [95], and p38 helps in binding the Atf4 factor to the CHOP promoter and proceeds in the expression of caspase-4 [96].

Several experimental studies have investigated the therapeutic efficacy of p38 MAPK as a drug target which manifested the role of p38 MAPK inhibitors against neuroinflammation. Pinocembrin, a natural flavonoid, has been used in a study that attenuated the cerebral cortex neuronal degeneration and ameliorated the memory and cognitive function in the AD mouse model. It also showed remarkable results in diminishing the p38 MAPK activation and NF-*κ*B signaling [97]. Another study reported the efficacy of an essential oil Z-ligustilide (Z-LIG) extracted from umbelliferous plants, which showed reduced levels of intracellular ROS and regulated the apoptosis in A*β*-induced toxicity in PC12 cells [98]. Moreover, it manifested promising results in protecting against neurotoxicity via the activation of PI3K/Akt and the regulation of the p38 MAPK pathway in differentiated PC12 and SHSY5Y neuroblastoma cells [98, 99].

#### 2.2.3. JAK-STAT Pathway

The signal transducer and activator of transcription (STAT) factors are provoked by several cytokines and growth factors [100], and the disease and age-dependent decline in the JAK2/STAT3 pathway plays a central part in the AD pathogenesis. These growth factors are involved in the modulation of several genes, caspases, and the cell cycle regulators, which can also counter oxidative stress [100], and these factors are phosphorylated at tyrosine residues in reaction to several cytokines and growth factors [101]. Phosphorylation of STAT leads to its dimerization, and it becomes capable of binding to DNA, whereas in an unphosphorylated state, STATs are located in the cytoplasm and transfer to the nucleus on activation [102]. For the treatment with interferons, the Janus kinase (JAK) is essential for the phosphorylation of STAT factors [100]. The STAT members are diverse in their function and are accounted to play a crucial role in differentiation, proliferation, and cell survival [90–103]. They function as essential mediators in cytokine receptor signaling and are involved in the modulation of transcriptional target genes. It has been investigated that inactivation of STAT3 is associated with the AD pathogenesis, and the specific antibody has been analyzed against the phosphorylated form of STAT3, which exhibited its reduced levels in hippocampal neurons in AD patients [104]. In the context of higher levels of A*β* soluble peptides observed both in sporadic and familial forms of AD, it is postulated that these toxic peptides are involved in the inhibition of STAT3 proteins. In addition, several other pieces of evidence support that activated STAT3 was found to be attenuated in hippocampal neurons in AD mice models [105], which proposes that A*β* levels are inversely proportional to the levels of activated STAT3 in the hippocampal neurons. These toxic peptides and STAT3 are having direct effects leading to the alteration of STAT3, which was confirmed from the in vitro study that A*β* induction persistently attenuated the STAT3 levels in primary hippocampal neurons [104]. Aging is also considered one of the factors leading to the inhibition of STAT3 in association with AD, as observed from the study that immunoreactivity of p-STAT3 in hippocampal neurons was found to be upregulated compared to the older subjects in animal and human models [104].

The JAK-STAT pathway acts as a possible therapeutic target to the toxicity, as it is involved in the microglial activation and the modulation of inflammatory responses [106, 107]. Cytokines and the ligand binding to their respective receptors enhance the action of tyrosine kinase of JAK, which leads to the activation of receptors by phosphorylating them and triggering STATs and their phosphorylation as well and its dimerization successively leads to the nuclear translocation and transcription function. Previously it has been documented that the JAK-STAT pathway is linked with inflammatory activities and has been targeted for therapeutic compounds, and it is under clinical trial [108]. Large amounts of A*β* contents are associated with the enhanced phosphorylation of tyrosine in animal and human models [109], whereas the JAK-STAT3 pathway regulation manifested the inactivation of microglia and astrocytes in animal models of AD [110].

**Figure 2:**
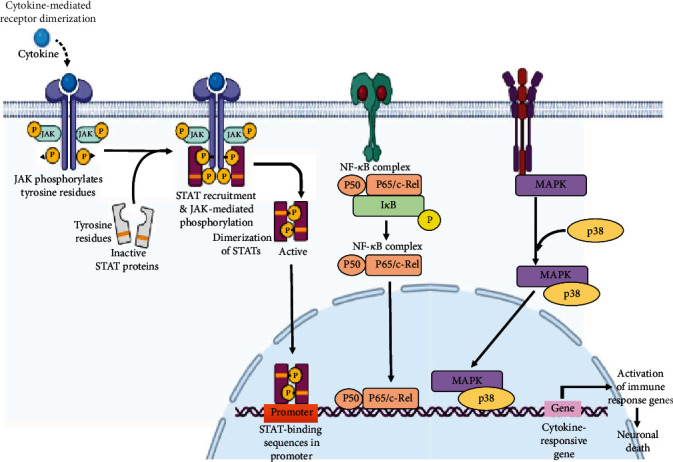
Inflammatory cytokines initiate the activation of the PI3K (phosphoinositide 3 kinase) pathway, phosphorylate the JAK-STAT (Janus kinases, signal transducer and activator of transcription proteins) factors, which activate the NF-*κ*B (nuclear factor kappa-light-chain-enhancer of activated B cells) pathway and enhance the production of ROS leading to apoptosis. p38 MAPK (a mitogen-activated protein kinase) is involved in the AD mechanism which includes the cytokine activation of p38 MAPK in microglia, leads to the increased production of proinflammatory cytokines which initiates the inflammatory process, and the cytokines also initiate the activation of p28 MAPK in astrocytes and neurons, which further escalates the inflammation. All these events lead to hyperphosphorylation, inhibition of long-term potentiation, apoptosis and synaptic dysfunction. NF-*к*B is a regulated transcription factor, involved in the regulation of inflammation, cellular growth, immune function and apoptosis. Free radical production led to the activation of IKB which phosphorylates the NF*к*B inhibitor, initiates the proteasomal degradation of IKB (IkappaB kinase) and the liberation of NF*к*B which translocates into the nucleus and binds to the DNA responsive element. Together with the coadjuvant and other activators, the increased expression of proinflammatory cytokines is triggered and neuroinflammation is supported, which causes the degeneration of neurons and eventually leads to the progression of AD (created with http://BioRender.com/).

Phytochemicals are being used extensively and have been implemented in the treatment of various disorders. They exhibit efficient therapeutic results against the diseases which gained the interest of scientists towards the plant-derived compounds and can target several pathways in the liberation of their therapeutic effects. Curcumin a natural flavonoid has been used in an experimental study that manifested the anti-inflammatory, antioxidant, cardioprotective, and neuroprotective effects [111–114]. Curcumin has shown promising results against various neurological diseases and proved to be effective in attenuating memory dysfunction and improved cognitive function [115]. Inflammation plays an influential role in the arrival of several disorders, which can be regulated by targeting the JAK-STAT signaling pathway [116]. Its inhibition shows positive results in diminishing the inflammation via attenuating the levels of inflammatory mediators [117]. Several phytochemicals such as curcumin, resveratrol, and epigallocatechin, etc manifest promising therapeutic results and anti-inflammatory response by attenuating the levels of inflammatory markers via targeting the JAK-STAT pathway [118–120] (Figure 2).

### 2.3. Other Mechanisms Driving Neuroinflammation and Their Potential Targets

#### 2.3.1. Activation of Oxidative Stress Pathway (Redox Signaling)

Oxidative stress is one of the factors responsible for the consequences of neuroinflammation. It is the mechanistic factor in the progression of inflammatory responses. Hydrogen peroxide and nitric oxide oxidants can come into existence either internally by initiating signal transduction processes or can get into the cell from the external environment. Cytokines are involved in the production of ROS via NADH oxidase (NOX) which is a ubiquitous mechanism commonly intimated for inflammatory factor-stimulated intracellular ROS production. The reduction of oxygen is catalyzed by NADPH oxidase to produce superoxide anion and is being released either at the surface of the cell or inside the cellular compartments [121]. Inducible nitric oxide synthase (iNOS) together with the microglia and astrocytes trigger the generation of nitric oxides. ROS/RNS perform significantly upon NO, thiols, and redox sensors. Cellular thiols react to produce S-nitrosothiols (RSNO) and sulfenic acids (RSOH), and both these contents undergo exchange with GSH to yield protein SSG species [122, 123], which is reprocessed to its active form. Glutathionylation is one regular content in redox signaling and ROS/RNS are also other mechanisms involved in redox signaling reacting with heme and transcriptional factors [123, 124]. The outcome of ROS/RNS initiated redox signaling in the neuroinflammatory motif is generally an increased expression of transcription factors that regulate the levels of chemokines, cytokines, metabolizing catalases, and ROS-producing enzymes. Cytokines are involved in the stimulation of iNOS in astroglia and microglial cells by directly venturing surface receptors leading to the generation of increased NO that may be toxic and become a threat to neurons. It has been investigated that AD brains exhibited higher production of iNOS and knockout of iNOS genetically has been observed to be protective against AD in mouse models [125]. Additionally, elevated levels of iNOS in AD have also been exhibited to be associated with NO-induced posttranslational modifications such as S-nitrosylation, nitration, and dityrosine generation [126]. At the tyrosine 10 residue, nitration of the A*β* peptide manifested the tendency of A*β* to accumulate and be recognized in the senile plaques [127].

#### 2.3.2. Activation of Complement System Pathways

The complement system is the principal component and indicates a fundamental pathway for the activation of immune reactions and provides defense against foreign harmful agents. This system is also activated in neurodegenerative disorders and the proteins included are linked with pathogenic lesions in AD. These complement proteins once activated lead to the opsonization and support the proteolysis of microorganisms. Astrocytes do take part in the production process but to a little extent, whereas the microglial cells generously lead the production of proteins of the complement system [128]. A study reported that functional complement proteins are linked with A*β* toxic deposits and also the A*β* peptides are playing a critical role in the activation of the complement system observed in in vitro studies through the alternative pathway [129]. Further, it was confirmed from the study that Clustrin a heterodimeric protein (apolipoprotein J), acts as a soluble inhibitor and the complement receptor-1 is involved in the progressing and removing of opsonized immune networks linked with AD, proclaiming the significance of the complement system in AD [130, 131].

#### 2.3.3. Activation of Cytokine and Chemokine Pathways

Cytokines are the key players that include proinflammatory and anti-inflammatory cytokines involved in the neuroinflammation process and are produced by both astrocytes and microglial cells. A study revealed that in aged transgenic mice, expansion of A*β* was observed which is associated with the upregulation of proinflammatory cytokines [132]. This study supports that A*β* is the key player that triggers neuroinflammatory responses in AD. Exposure of microglia to A*β* promotes the activation of proinflammatory cytokines and macrophage colony-stimulating factor (M-CSF) [133]. In the CNS and plasma of AD patients, contents of M-CSF were depicted in an elevated state compared to healthy subjects [134, 135]. A subsequent higher level of IL-1*β* was observed in AD patients, and the activation of caspase-1 is essential for the activation of IL-1*β*, which has been found in an increased state in MCI and AD patients [136]. TNF-*α* is considered to be the most critical proinflammatory factor in AD, which promotes and modulates the cytokine members during the process of inflammation [137]. It can expand the endothelial adhesion proteins and directs the immune cells to get recruited in the defective area [138]. It is confirmed from the study that in the plasma and brain tissues of Also, TNF-*α* can play an instrumental role in enhancing the A*β* elements via the enhanced production of *β*-secretase and advanced activity of the *γ*-secretase enzyme [139, 140]. Chemokines are involved in the regulation of microglial movement to the affected areas by the process of neuroinflammation in AD, associated with the development of CNS, and are communication factors in between the cells [141]. The CCL2 chemokine and its receptor play a critical part in controlling the crossing over of peripheral monocytes inside the brain, which confirms its purpose in AD [141]. There is an increase in the activity of chemokine CCL2 and the receptors CCR3 and CCR5 in reactive microglial cells, and there is a depiction of chemokine CCL4 in astrocytes around A*β* plaques [142]. It has been demonstrated that the induction of A*β* upregulated the levels of CXCL8, CCL2, and CCL3 while assessing the microglial cultures procured from the autopsies of AD [143]. Moreover, CCR2 and CCR5 receptors have potential to attenuate the advancement of the disease by impacting the function of microglial cells [144, 145].

#### 2.3.4. Activation of Cyclooxygenase Pathways

Cyclooxygenases (COX) are involved in proinflammatory responses, and their expression is upregulated when inflamed and provoked in cells and tissues, which play a significant role in the generation of prostanoids [146]. It helps in the conversion of arachidonic acid into prostaglandinH2 (PGH2), where the prostanoids begin to synthesize with varied functions. COX-2 acts as a regulating factor in moderating the synaptic transmission localized at the post-synaptic sites but it has been documented that overexpression during pathological conditions may trigger neuronal damage and cognitive dysfunction [147]. Moreover, the excessive activation of COX-2 in neurons may trigger the expansion of A*β* generation and cognitive impairment has been investigated from various mouse models [148, 149]. This finding supports the activities of COX-2 and its outcomes in AD.

#### 2.3.5. Opening of Blood-Brain Barrier with Aging

**Figure 3:**
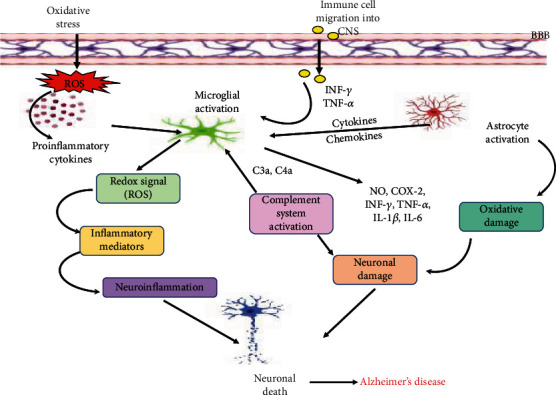
Other mechanisms driving neuroinflammation: increased oxidative stress either by excessive production and release of ROS (reactive oxygen species) of inflammatory mediators leading to the overproduction proinflammatory cytokines. Proinflammatory factors activate the glial cells and promote the process of neuroinflammation. Several antioxidants including SOD (superoxide dismutase), Cat (catalase), and GPx (glutathione peroxidase) may act as reducing agents in attenuating ROS production and diminish the inflammatory response. Activated glial cells under the influence of several proinflammatory cytokines trigger the complement system, and the released cytokines form T cells. Activated glial cells further promote the release and activation of inflammatory cytokines such as TNF-*α* (tumor necrosis factor), IL-1*β* (interleukin-1*β*), IL-6 (interleukin-6), NO (nitric oxide), COX-2 (cyclooxygenase-2), IFN-*γ* (interferon gamma), and chemokines which cause damage to the neurons and lead to their degeneration.

The blood-brain barrier (BBB) functions as a main defensive factor in the periphery of the brain which restricts the unspecific elements to make entry into the brain and inhibits the passaging of cells and molecules in between blood and brain tissues. During AD events, BBB becomes deficient and loses the selective and defensive function, as noticed from the inflammatory responses in the CNS at the site of injury and systemic infection leads to the disintegration in dementia [150, 151]. However, in normal conditions, BBB efficiently passes the A*β* into the blood and restricts the entry of A*β* from the serum into the CNS, whereas all these normal events are manipulated and disrupted during the progression of AD. A*β*42 peptide is involved in the alteration of the tight junction among endothelial cells of the BBB, making them deficient and punctured which simply causes the accumulation of A*β* fibrils and produces further burden and toxicity to the BBB. Moreover, proinflammatory cytokines such as TNF-*α* and IL-1*β* also take part in the distraction of tight junctions which are produced in excessive quantity by microglia in AD conditions [152], which makes the easy entry of macrophages and T cells in the CNS. Thus, these additional cells cause the extra alteration of astrocytes and microglial cells to initiate the inflammation, which leads to the additional liberation of inflammatory markers (Figure 3).

## 3. Role of Inflammation on Tau Pathology in AD

**Figure 4:**
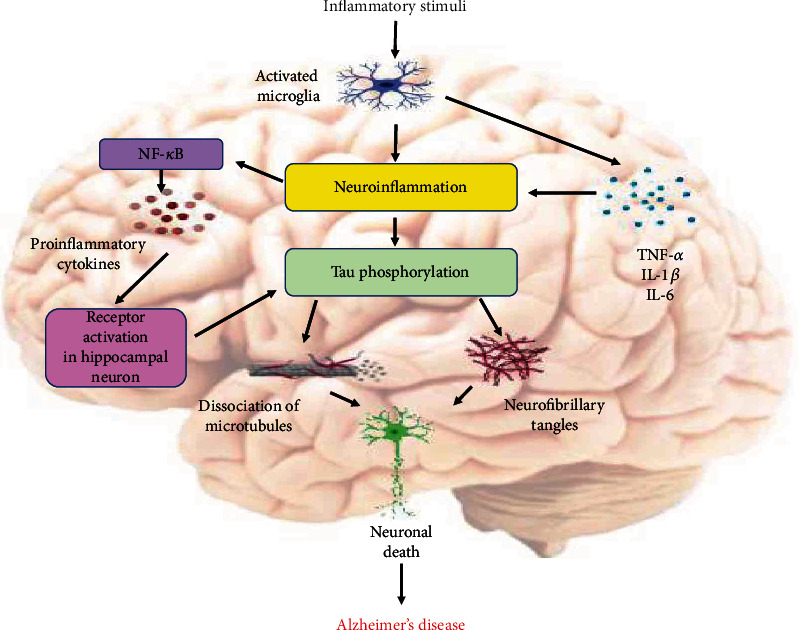
Role of inflammation on Tau pathology: inflammatory stimuli activate the microglial cells and trigger production of proinflammatory cytokines and Tau accumulation in the AD brain. Proinflammatory mediators such as TNF-*α* (tumor necrosis factor), IL-1*β* (interleukin-1*β*), and IL-6 (interleukin-6) could trigger neuroinflammation and tau pathology. Neuroinflammatory response activates a signaling cascade with the release and activation of NF-*κ*B (nuclear factor kappa-light-chain-enhancer of activated B cells), overproduction of proinflammatory cytokines, and the activation of neuronal receptors. Hyperphosphorylation of tau protein initiates the dissociation of microtubule. Soluble tau aggregates into pathological tau oligomers, forms tau filaments, and ultimately leads to the formation of neurofibrillary tangles, which promote the neuronal death (created with http://BioRender.com/).

Inflammation is explicated as a serious event and plays an instrumental role in tau pathology. Around the toxic lesion in the brain, the appearance of activated astrocytes and microglial cells, and the enhanced expression of inflammatory markers indicate the interrelation between the AD pathology and inflammation [47]. A study was conducted on P301S mutant tau transgenic mice which manifested that the microgliosis and synaptic pathology may be the early events of neuronal dysfunction linked to tauopathy [153]. Excessive activation of microglial cells may play a role in the formation of tangles, while the attenuated tau pathology has been observed in the transgenic mice via immunosuppression, which highlights that neuroinflammation is the key event in the advancement of tau pathology [153]. There are several means such as physical or chemical injury or infections caused by pathogens, which trigger inflammation in the brain regions and leads to the direct involvement and activation of astrocytes, microglial cells, and inflammatory cytokines. Inflammatory processes and oxidative stress in AD lead to the activation of several kinases including MAPK, GSK-3*β*, and cdc2 [23]. An experimental study revealed that chronic induction of LPS subsequently triggered the phosphorylation of tau protein at various sites related to tau pathology in transgenic mice [154]. Also, the stimulation of microglial cells and IL-1*β* liberation were engaged via the CDK-5 or GSK-3*β* kinase activation [155]. The persistent activation of TNF-*α* has also been found to be associated with the formation and increase in the production of pre-tangle-linked pT231 epitope [156]. Proinflammatory cytokine interferon-*γ* (IFN-*γ*) plays a crucial role against viral infections, however, the increased expression of this cytokine was observed in a study that leads to the dephosphorylation of tau protein at phosphorylation sites [157]. Thus, confirms that the activation or inhibition of tau pathologies are linked to the proinflammatory cytokines and the activated glial cells (Figure 4).

### 3.1. Activation of Inflammasome

The number of pattern recognition receptors (PRRs) are provoked by various cell lineages inside the blood to detect and fight against pathogens [158]. PRPs are classified into two types based on their intracellular localization including Toll-like receptors (TLRs) and c-type lectin receptors (CLRs) which are localized in the cellular membrane and endosomes where they identify extracellular pathogen-associated molecular patterns (PAMPs) and danger-associated molecular patterns (DAMPs). A subset of PRPs contains NOD- (nucleotide oligomerization domain-) like receptor (NLR) and AIM2- (absent in melanoma 2-) like receptor (ALR) proteins, which can congregate multimolecular intracellular complexes called inflammasomes, which in turn can generate potent inflammatory reactions in response to stress and cellular contaminations [158, 159]. In an ATP-dependent manner, oligomerization of the NOD domain promotes activation of the inflammasome. The inflammasome contains nucleotide-binding and oligomerization domains and a precursor of procaspase-1 [158].

Several inflammasomes have been recognized such as NLRP1, NLRP3, IPAF, and AIM2, among them, NLRP3 is the most extensively studied inflammasome comprised of the ASC adaptor, NLRP3 scaffold, and procaspase-1 [158]. This inflammasome participates in metabolic as well as inflammatory disorders including diabetes and neurodegenerative disorders (160). It has been manifested that A*β* protein aggregation may trigger the initiation of NLRP3 in microglial cells [160, 161], which may promote the production of activated IL-1*β* and caspase-1. Further, it has been evidenced that microglial cells in animal models and AD subjects may exhibit enhanced levels of IL-1*β* and caspase-1 [136, 161, 162]. It has been documented that amyloid plaques instigate microglial cells to scavenge the A*β*-oligomers, which in turn triggers NLRP3 activation with a subsequent release of proinflammatory markers such as (IL-1*β* and IL-18) and several other neurotoxic factors. This process of liberation augments the neurotoxic effects of A*β* and aggravates the pathological processes of AD [161, 163–165]. Microglial cells are the prime components in the system which play a critical role in the inflammatory responses, but the abnormal microglia-specific NLRP3 activation may trigger chronic neuroinflammation [166].

## 4. Role of Inflammation on A*β* Pathology in AD

**Figure 5:**
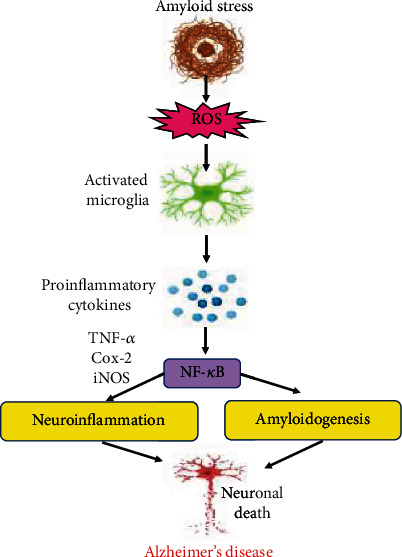
Role of inflammation on A*β* pathology: A*β* stress leads to the production of ROS, and the inflammatory stimuli activate the microglial cells, which leads to the production of proinflammatory cytokines and causes the elevated levels of cytokines and accumulation of activated microglia. The promoter region of NF*к*B (nuclear factor kappa-light-chain-enhancer of activated B cells) has binding sites, which lead to the process of amyloidogensis and inflammation, and the activated NF*к*B initiates the production of proinflammatory cytokines, which triggers neuroinflammation. These mediators cause the excitation of the glial cells which further stimulate the production of A*β* burden and additional proinflammatory cytokines and ultimately leads to the death of neurons and AD pathology.

Inflammation is believed to be the prime cause of AD, and the deposition of toxic proteins inside the brain trigger inflammatory responses which lead to the aggravation of the disease. Injured or damaged regions of the brain tissues initiate inflammation, which is provoked by the AD toxic lesions and subsequent neuronal damage. The inflammatory process triggers the implementation and functioning of several inflammatory markers in response to injury or by other toxic events which are involved in inflammation. Early A*β* oligomer deposition in the brain may promote the activation of glial cells, enhanced levels of cytokines, and activation of the complement system [167]. Following the accumulation of A*β* peptides, an immune reaction in the brain triggers the release and activity of inflammatory markers such as TNF-*α*, IL-1, IL-6, and the activation of specialized brain cells [168, 169] The inflammatory process takes place in the brain having dense and abridged pathologies of AD, though there are minor cases of inflammation in the brain regions with reduced AD pathologies [25]. Transgenic animals were observed in an experimental study that depicted their execution of inflammatory cytokines which triggered several pathological alterations such as neuronal damage, demyelination, and activation of astrocytes and microglial cells [30]. A*β* production and its clearance are the two main events in the progression of AD, in which the ineffective clearance of A*β* in the brain leads to the accumulation of the toxic peptides that are associated with AD, although, microglia could help in the eviction of A*β* at the beginning and play an indispensable role in the inhibition of AD pathology. Hence, all these pieces of evidence support that inflammation is the key process in the AD pathology by which several proinflammatory cytokines enhance A*β* generation and the malfunction of specialized cells inside the brain, diminishing the levels of A*β*-degrading enzymes, and damaged BBB (Figure 5).

## 5. Prevention Is Better than Cure

Presently, no pharmacological therapy has been elucidated to be completely curable for AD. From the past few years, scientists, researchers, and the public have gained much interest in phytochemicals, as they proved to be effective in preventing various neurodegenerative diseases. For decades, traditionally acquired medicines from plants have been constantly implemented in the health care system in underdeveloped countries [3, 170]. Plant-derived medicines are in abundance with less toxicity and minimal health hazards and are more economical than synthetic drugs [171]. For various ailments, medicinal plants are being extensively used in traditional therapy since phytomedicines exhibit psychotropic, adaptogenic, and neuroprotective properties.

Phytochemicals possess anti-inflammatory, antioxidant, antiapoptotic, and neuroprotective properties and are broadly disseminated in the plant kingdom with minimum adverse effects [152, 172, 173]. Researchers have demonstrated that phytochemicals including nutraceuticals played a significant therapeutic role against AD. It has also been evidenced that the antioxidants vitamin C and E played a potential role against oxidative stress in AD patients, but more studies are needed to further know the detailed approaches of antioxidants [174, 175]. Several phytochemicals including curcumin, thymoquinone, lycopene, piperine, anthocyanins, and catechins used in both in vitro and in vivo animal models manifested anti-inflammatory and antioxidant properties [176–178]. In AD, neuroinflammation leads to a significant increase in the expression of inflammatory cytokines, including TNF-*α*, IL-6, and IFN-*γ*, and alterations in the levels of oxidative markers [41, 179, 180]. Researchers reported that supplementation of lycopene had no influences on peroxidation markers and cytokines in asthmatic and obese patients, but supplementation with polyphenols showed marked results in patients. However, green tea extract significantly ameliorated the inflammatory and oxidative markers in obese as well as in hemodialysis patients. Likewise, curcumin attenuated IL-1*β* and peroxidation markers in osteoarthritis subjects but showed no changes in obese patients.

The intervention of curcumin, grape juice, blueberry, coca flavanols, and green tea extracts ameliorated cognitive function in humans as well in mouse models [181, 182]. Polyphenolic nutraceuticals have been reported to provide neuroprotection through the modulation of misfolded amyloid-beta and hyperphosphorylated tau protein generation [183], initiation of inflammatory cytokines (TNF-*α* and IL-1*β*), modulation of MAPK and NF-*κ*B pathways, and inception of nuclear factor-erythroid 2-related factor 2 (Nrf2) or antioxidant responsive elements. All these events lead to the activation of several enzyme cascades to deliver antitoxic and antioxidant properties [184]. Several phytochemicals from herbs, spices, and extracts of medicinal plants including terpenoids and triterpenoids trigger the modulation of NF-*κ*B or initiation of Nrf2 nuclear factor [185, 186]. Several studies reported that antioxidant beverages and apple and green tea extracts have significantly attenuated the TNF-*α* and IFN-*γ* proinflammatory cytokine expression in AD patients at the initial stage, whereas expression levels of these cytokines were significantly upregulated at the moderate stage of AD [187]. However, IL-1*α* is in an increased level in the moderate stage of AD, but no significant changes were observed in the initial stage of AD, although antioxidant beverages also did not cause any significant improvement or alteration in the levels of IL-1*α* in both of the stages of AD [187]. Therefore, it is demonstrated that nutraceutical-provoked interactions between I*κ*B, I*κ* kinases, and electrophiles lead to the modulation of NF-*κ*B and Nrf2 factors [188–190].

It is evidenced from several studies that flavonoid metabolites may play an essential role and serve as a precursor of drugs, thereby attenuating the generation of ROS and secretion of inflammatory cytokines [136, 156–194]. Although, naringenin flavonoids do not always trigger antioxidant and anti-inflammatory responses, naringenin-4-O-glucuronide, a predominant metabolite of naringenin, upregulated the expression of TNF-*α* and Nrf2 whereas other metabolites of naringenin-7-O-glucuronide attenuated the expression of Nrf2 factor [136, 158–195]. The blood-brain barrier (BBB) is the semipermeable membrane that inhibits nonselective crossing of solute into the CNS extracellular fluid and separates the brain from the periphery. It has been speculated that antioxidant and oxidative stress markers may not be detected inside the brain [196]. However, it has been observed in experimental studies that flavonoids do cross the BBB upon the oral induction of flavonoids to rodents, thereby allowing interaction; they were transported inside the brain by specific transporters expressed in the BBB [182, 197]. Various studies manifested that the ATP-binding cassette (ABC) induces multidrug resistance (MDR), and MDR transporters may contribute to AD pathology; the accumulation of A*β* fibrils in the brain [198] targeting these ABC transporters are being considered as the biomarkers for the amelioration of AD pathology [199]. Hence, it has been observed that acute consumption and induction of flavonoids may play a vital role in the modulation of MDR, via the activation protein-1 (AP-1), Nrf2, and NF-*κ*B pathways [200]. Thus, flavonoids are considered an efficient therapeutic option and may play an essential role in the prevention of AD.

A study reported that the extract of turmeric combined with green tea and black pepper extract showed promising results against AD [201]. It has also been reported that piperine exhibited antioxidant and anti-inflammatory properties and offered neuroprotection, thus acting as a preventive agent against AD. Although its bioavailability is low because of internal metabolism and having hydrophobicity, the employment of a nanoparticle-mediated delivery system augments its bioavailability to be accessible as a suitable vehicle for the prevention of AD [202]. Curcumin is another herbal supplement that is effective against AD, thereby attenuating the inflammation-associated markers in AD. Further, it was revealed that curcumin plays a promising role in diminishing the A*β*-provoked neuroinflammation via PPAR*γ* activation in AD rat models [203]. Curcumin has also been found to be actively associated with astrocyte and microglial regulation, and modulated the NF-*κ*B pathway, as well as reduced the production of proinflammatory cytokines [204]. Having less bioavailability, it is also being encouraged to reach the desired site via executing several nanoparticles, thereby playing a significant role in the prevention of AD [205].

Curcumin has been reported to trigger the stimulation of BDNF and regulate the extent of TNF-*α*, NF-*κ*B, and caspase-3. It has been shown to activate the ERK/PKC and Akt/GSk3*β* signaling pathway in a D-galactose-induced mouse model [206]. Epigallocatechin-3-galate has been found to regulate the initiation of NF-*κ*B and MAPK pathways and reduce the levels of IL-1, IL-6, IL-8, and COX-2 inflammatory markers. It also exhibited the secretion of BDNF and NGF and reduced the levels of caspase-3 and ROS in human astrocytoma U373MG cells [207]. The naringenin compound was reported to promote the activation of Nrf2/ARE signaling, augment the levels of antioxidants, and reduce the extent of NO, cytokines, and NF-*κ*B signaling in the hypoxia rat model [208]. The *α*-mangostin compound has been found to play an instrumental role in the regulation of inflammatory responses, augment the expression of BDNF protein, and reduce the phosphorylation of tau protein. It has also been shown to regulate the levels of inflammatory markers including IL-1*β*, TNF-*α*, and caspase-3 in the C57BL/6J triple transgenic mouse model [209]. It is manifested that Asiatic acid, a triterpenoid of *Centella asiatica*, diminished the expression of APP, A*β*1-42, *β*, and *γ*-secretases. Also, it has been found to attenuate the expression of inflammatory mediators in the hippocampus and cortex regions of the brain and enhance the expression of GFAP and Iba-1 proteins in the aluminium-induced AD rat model [99]. Thymoquinone, an extract of *Nigella sativa* was shown to inhibit the NF-*κ*B triggered neuroinflammation and subsequently suppress the production of (NO, IL-1*β*, and TNF-*α*) inflammatory mediators by regulating the PI3K/Akt/NF-*κ*B signaling pathway in LPS/IFN-*γ* activated BV-2 microglia [210]. Gingerol, a compound of *Zingiber officinale*, has been documented to ameliorate the cognitive, behavioral deficits, and AD-like pathology. It enhances the *α*-secretase activity and reduces cerebral A*β*-42-, *β*-secretase-, and COX-2-associated neuroinflammation in the ICV-STZ-induced mouse model [211]. Researchers revealed that hesperidin inhibits the stimulation of (TNF-*α*, IL-1*β*, COX-2, and iNOS) inflammatory mediators. It enhances the antioxidant defense, augments the cognitive function, and attenuates the A*β* pathology by reducing H_2_O_2_ levels and restoring reduced GSH levels and total antioxidant capacity in the A*β*-induced mouse model [212]. Quercetin is another phytochemical extracted from grape vine reported to provide protection to neuronal cells by diminishing oxidative stress and neuroinflammation. It has also been revealed that quercetin inhibits A*β* aggregation and tau hyperphosphorylation and regulates the neuroinflammatory process by reducing the levels of iNOS, COX-2, and IL-1*β*, and diminishes the levels of GFAP in the SAMP8 senescence model [213] (Table 1).

## 6. Conclusion and Future Prospective

Based upon the previous studies, it has been elucidated that inflammatory responses are the major cause and have been highly involved in the progression of AD. Biomarkers for the disease play an influential role in the earlier detection and intervention of pharmacotherapy for the effective prevention of inflammatory responses and the development of selective inhibitors that spot specific arbitrators in the inflammatory cascade. Astrocytes and microglial cells perform integral roles during the development and physiology of the brain. Astrocytes are involved in the maintenance of the CNS, which offers structural stability, receptive surface insulation, and cushioning of extracellular chambers. These supportive cells play a significant role against inflammation and proceed to divide and block impaired areas. Synaptic plasticity and synaptic transmission are being modulated by astroglial cells, thereby providing protection to neurons against toxicity, and metabolically deliver enough support to warrant their effective performance. In addition, astrocytes are also involved in the inception and the advancement of AD.

Several anti-inflammatory drugs have been administered against AD models, but to date, the anti-inflammatory drugs have not exhibited comprehensive protection against the disease. More studies are warranted to determine the anti-inflammatory responses and the use of the appropriate model to facilitate the positive effects of therapeutic drugs. In recent times, phytochemicals have intrigued and drawn enough attention for the development of new therapeutic drugs for the prevention of AD. The therapeutic approaches of polyphenolic compounds, nutraceuticals, and antioxidants have been well elucidated and have shown positive results against neuroinflammation in AD and various other neurodegenerative diseases. Researchers believe that phytochemicals and polyphenols may revive the AD pathologies as they have shown promising results against the disease and may represent as excellent preventive agents against AD by modulating pathways related to inflammation. Several polyphenols have been studied for therapeutic approach and efficiently exhibited positive results and are entertained as the best preventive agents against AD. However, more studies are required to develop phytochemicals as novel preventive vehicles for AD.

## Figures and Tables

**Table 1:** Phytochemicals that affect neuroinflammation in animal and cellular AD models.

Name of the plant	Phytochemicals	Experimental model used	Mechanism of action	References
Turmeric, *Curcuma longa*	Curcumin	SQ-injected D-galactose-induced mouse model	It activates ERK/PKC-arbitrated CREB regulation and Akt/GSk3*β*-arbitrated regulation. Stimulates BDNF and regulates the levels of caspase-3, TNF-*α*, and NF*к*B	[206]
Tea plant, *Camelia sinensis*	Epigallocatechin-3-galate	Human astrocytoma U373MG cells	It regulates the activation of NF*к*B and MAPK; reduces the levels of IL-1, IL-6, IL-8, and Cox-2; promotes the secretion of BDNF and NGF; attenuates caspase-3 and ROS levels	[207]
Sweet orange, *Citrus sinensis*	Naringenin	Hypoxia rat model	It initiates the activation of Nrf2/ARE signaling; enhances the levels of antioxidants; attenuates the levels of NO, cytokines, and NF*к*B signaling	[208]
Mangosteen, *Garcinia mangostana*	*α*-Mangostin	C57BL/6J triple transgenic mouse model	Plays an essential role in the regulation of inflammatory process; enhances BDNF expression and attenuates the phosphorylation of tau; regulates the levels of IL-1*β*, TNF-*α*, and caspase-3	[209]
Indian pennywort, *Centella asiatica*	Asiatic acid	Aluminium-induced rat model	Asiatic acid attenuates the A*β* toxicity by reducing the levels of APP, A*β*1-42, and *β*- and *γ*-secretases. It also reduces the expression of inflammatory mediators in the hippocampus and cortex and enhances the expression of GFAP and Iba-1	[99]
Black seed, *Nigella sativa*	Thymoquinone	LPS/IFN-*γ*-activated BV-2 microglia	Inhibition of NF*к*B initiated neuroinflammation, suppression of inflammatory markers (NO, IL-1*β*, and TNF-*α*), and production by regulating PI3K/Akt/NF-*к*B signaling	[210]
Ginger, *Zingiber officinale*	Gingerol	ICV-STZ-induced mouse model	It ameliorates the cognitive and behavioral dysfunction and AD-like pathology. It enhances the *α*-secretase activity and attenuates cerebral A*β*-42, *β*-secretase, APH1a activity, and COX-2-associated neuroinflammation	[211]
Citrus, *Citrus* × *sinensis*	Hesperidin	A*β*-induced APPswe/PS1dE9 transgenic mouse model	It exhibits the inhibitory effect on inflammatory mediators (TNF-*α*, IL-1*β*, COX-2, and iNOS). It enhances antioxidant defense and improves cognitive function. It attenuates the A*β* pathology by reducing H_2_O_2_ levels and restoring depleting GSH levels and total antioxidant capacity	[212]
Grape vine, *Vitis vinifera*	Quercetin	SAMP8 (senescence model)	It protects neuronal cells by reducing oxidative stress and neuroinflammation. It inhibits A*β* aggregation and tau phosphorylation. It suppresses neuroinflammatory processes by decreasing proinflammatory cytokines (iNOS, COX-2, and IL-1*β*) and reduces the levels of GFAP in the hippocampus	[213]
